# Supplementary material for: The Effect of Socio-Demographic Factors in Health-Seeking Behaviors among Bangladeshi Residents during the First Wave of COVID-19
Source: Healthcare (Basel). 2022 Mar 4;10(3):483. doi: 10.3390/healthcare10030483 (PMC8949002; doi:10.3390/healthcare10030483)
Supplement: Supplementary file 1 [file healthcare-10-00483-s001.zip › healthcare-1580006-supplementary.pdf]

healthcare

MDPI

Table S1. Health seeking behavior score among the different demographic characteristics of the study participants.

Age	Overall (n = 947)	Male (n = 568)	Female (n = 379)
11-20 years	9.31 ± 2.12	9.44 ± 2.27	10.2 ± 1.73
21-30 years	10.2 ± 1.86	9.74 ± 1.87	10.8 ± 1.65
31-40 years	9.68 ± 2.20	9.20 ± 2.14	10.4 ± 2.08
41-50 years	9.12 ± 2.23	8.90 ± 2.26	9.58 ± 2.14
>50 years	9.96 ± 2.45	9.70 ± 2.25	10.2 ± 3.03
**Education**			
Illiterate	6.93 ± 1.49	6.81 ± 1.64	7.09 ± 1.30
Primary	7.55 ± 1.69	7.43 ± 1.74	8.18 ± 1.33
SSC	8.68 ± 2.09	8.29 ± 2.17	9.14 ± 1.96
HSC	10.1 ± 2.44	9.17 ± 2.20	12.4 ± 1.09
Undergraduate	10.1 ± 1.72	9.76 ± 1.74	10.6 ± 1.60
Post graduate or above	10.5 ± 1.76	10.2 ± 1.76	11.0 ± 1.64
**Occupation**			
Government Job	10.4 ± 1.77	10.0 ± 1.74	11.12 ± 1.60
Private Job	10.32 ± 1.75	10.0 ± 1.80	10.9 ± 1.52
Business	8.46 ± 2.04	8.19 ± 1.77	10.2 ± 2.95
Daily wager[a]	7.08 ± 1.58	6.94 ± 1.51	8.0 ± 1.87
Home maker	9.36 ± 2.36	-	9.36 ± 2.36
Unemployed	10.1 ± 1.93	9.80 ± 1.90	10.7 ± 1.87
Others[b]	9.98 ± 2.03	9.30 ± 2.13	10.6 ± 1.68
**Monthly income**			
<10,000 BDT	9.77 ± 2.14	9.27 ± 2.09	10.4 ± 2.01
10,000-30,000 BDT	9.83 ± 2.12	9.33 ± 2.27	10.5 ± 1.67
>30,000 BDT	10.4 ± 1.59	10.1 ± 1.55	11.2 ± 1.43
**Locality**			
Slum	8.18 ± 2.34	7.81 ± 2.30	9.00 ± 2.30
Modern residential area	10.5 ± 1.88	10.1 ± 1.93	11.2 ± 1.58
Common housing area	9.88 ± 1.95	9.43 ± 1.93	10.5 ± 1.82

BDT= Bangladeshi Taka; [a]rickshaw puller, day laborer, etc.; [b]house tutor, part-time, etc.; [c]village, hostel, dormitory, etc.

Table S2. Association of socio demographic factors and predicting health seeking behavior during the pandemic stratified by sex.

Variables	Overall (n = 947)		Male (n = 568)		Female (n = 379)	
	β-(95% CI)	p-value	β-(95% CI)	p-value	β-(95% CI)	p-value
**Age**						
>50 years	Ref.		Ref		Ref	
11-20 years	-0.76(-0.20, -0.96)	0.010	0.01(-0.71, 0.73)	0.977	-0.07(-1.06, 0.93)	0.894
21-30 years	-0.34(-0.80, 0.13)	0.154	-0.33(-0.91, 0.25)	0.300	-0.03(-0.86, 0.80)	0.938
31-40 years	-0.28(-0.74, 0.19)	0.241	-0.39(-0.97, 0.19)	0.191	0.04(-0.79, 0.87)	0.923
41-50 years	-0.30(-0.83, 0.23)	0.273	-0.34(-1.00, 0.32)	0.309	-0.03(-0.99, 0.93)	0.949
**Education**						
Illiterate	Ref.		Ref.		Ref.	
Primary	0.63(-0.17, 1.43)	0.125	0.62(-0.38, 1.61)	0.225	1.09(-0.25, 2.43)	0.111
SSC	1.75(0.83, 2.67)	<0.001	1.48(0.26, 2.71)	0.018	2.05(0.78, 3.32)	0.002
HSC	3.18(2.34, 4.02)	<0.001	2.36(1.30, 3.42)	<0.001	5.34(4.07, 6.61)	<0.001
Undergraduate	3.19(2.49, 3.89)	<0.001	2.95(2.04, 3.87)	<0.001	3.48(2.50, 4.47)	<0.001
Post graduate or above	3.61(2.91, 4.30)	<0.001	3.36(2.45, 4.27)	<0.001	3.94(2.97, 4.92)	<0.001
**Occupation**						
Unemployed	Ref.		Ref.		Ref.	
Government Job	0.28(-0.12, 0.67)	0.169	0.21(-0.29, 0.70)	0.412	0.44(-0.16, 1.04)	0.150
Private Job	0.18(-0.14, 0.50)	0.278	0.21(-0.19, 0.62)	0.304	0.20(-0.16, 1.04)	0.150
Business	-1.68(-2.33, -1.03)	<0.001	-1.61(-2.31, -0.90)	<0.001	-0.48(-2.08, 1.11)	0.552
Daily wager[a]	-3.07(-3.70, -2.44)	<0.001	-2.85(-3.53, -2.17)	<0.001	-2.68(-4.28, -1.09)	0.001
Home maker	-	-	-	-	-1.33(-2.06, -0.59)	<0.001
Others[b]	-0.18(-0.54, 0.18)	0.317	-0.58(-1.07, -0.09)	0.021	-0.04(-0.54, 0.46)	0.881
**Monthly income**						
<10,000 BDT	Ref.		Ref.			
10,000-30,000 BDT	0.06(-0.15, 0.28)	0.559	0.07(-0.35, 0.49)	0.751	0.10(-0.35, 0.54)	0.676
>30,000 BDT	0.66(0.46, 0.86)	<0.001	0.83(0.45, 1.21)	<0.001	0.73(0.28, 1.19)	0.002
**Locality**						
Slum	Ref.		Ref.			
Modern residential area	1.27(0.82, 1.73)	<0.001	2.28(1.48, 3.08)	<0.001	2.21(1.15, 3.27)	<0.001
Common housing area	0.97(0.53, 1.41)	<0.001	1.62(0.85, 2.39)	<0.001	1.48(0.44, 2.51)	0.005
Others[c]	0.80(0.25, 1.34)	0.004	1.55(0.65, 2.23)	0.001	0.92(-0.51, 2.34)	0.207

Linear regression was used to estimate the p-value. BDT= Bangladeshi Taka; [a]rickshaw puller, day laborer, etc.; [b]house tutor, part-time, etc.; [c]village, hostel, dormitory, etc.
